# Client satisfaction with abortion care service and its associated factors among women in Ethiopia: a systematic review and meta-analysis

**DOI:** 10.1186/s12905-024-03139-3

**Published:** 2024-05-14

**Authors:** Temesgen Geta, Eskinder Israel, Christian Kebede

**Affiliations:** 1 School of nursing, Wolaita Sodo University, Wolaita, Ethiopia; 2 School of Public Health, Wolaita Sodo University, Wolaita, Ethiopia

**Keywords:** Abortion care, Women, Client satisfaction, Ethiopia, Systematic review and meta-analysis

## Abstract

**Background:**

The client’s satisfaction after abortion care is the key to sustaining abortion care and increasing the health status of those who had complications from abortion. Nevertheless, research conducted in Ethiopia stated that the major problem is the need for post-abortion care for females. One of the ways to improve the qualities involved in post abortion care and decrease the mortality and morbidity rates caused by unsafe abortion is by ensuring client satisfaction with abortion care. Strategy making and policy formulation based on systematic review take on the highest priority in developing countries. However, although some independent studies had been carried out in Ethiopia, their findings might not have been representative and conclusive. The main purpose of this systematic review and meta-analysis article is to establish the proportion of abortion clients who are satisfied with their abortion care and the factors that contribute to such satisfaction among clients in Ethiopia.

**Methods and materials:**

Only published articles were considered in this review. The main databases included Medline/Pubmed, Web of Science, Embase, Cinael, Med Nar, Google Scholar, Scopus, the Ethiopian University Repository Online, and the Cochrane Library. The review includes cross-sectional studies that meet the requirements and were written in English. A random effects model was used to calculate the pooled prevalence of client satisfaction with abortion care. The study heterogeneity was tested using Cochrane Q-Static and I2. Publication bias was checked using the Eggers test and funnel plot. PRISMA was used to select and direct the selection of articles for this review. Statistical analyses were conducted using STATA version 14.

**Result:**

A review of ten studies comprised 2740 women. In summary, the pooled prevalence of client’s satisfaction with abortion care in Ethiopia was 56.13% [95% CI (42.35; 69.91), I2 = 99.1%, *p* < 0.001]. In terms of subgroup analysis, Gambella had the highest prevalence of client satisfaction with abortion care at 87.40% [95% CI: 82.38 and 91.82]. However, Amhara had the lowest: 25.00% [95% CI: 21.59, 28.41]. The review also found that client satisfaction with abortion care had a statistical correlation with the type of procedure [OR: 0.25, CI [0.07, 0.42], I2: 76.9%, *p*-value: 0.041] and the participant’s education level [OR: 0.29, CI [0.09, 0.48], I2: 80.4%, *p*-value: 0.006].

**Conclusion:**

This review found that 56% of Ethiopian women were satisfied with their abortion care. Since this requires a boost to the quality of abortion care in the health facility, understanding women’s expectations and perceptions, training of health care providers, and strict monitoring of the quality of abortion care services by stakeholders like the Ethiopian government, non-governmental organizations, and high-level management of the health facility would help to improve the level of women’s satisfaction with abortion care. Those factors, namely, the type of method to use for the patient and women’s educational level, should be changed through improving awareness among the patients about what procedure to conduct and the health education provided to women about abortion care.

## Introduction

Overall, around 287,000 deaths of women occurred due to different issues resulting from pregnancy and labor worldwide in the year 2020 [1]. This indicated that maternal mortality is strikingly high internationally. More than two hundred thousand mothers in sub-Saharan African countries die from it, which accounts for 70% of all maternal moralities. The majority of deaths among pregnant women were linked to five major complications, such as unsafe abortion, postpartum hemorrhage, sepsis, and hypertension, among others, and obstetric difficulties. Nonetheless, most of these complication cases can be prevented or treated, but if not, they can cause maternal mortality or morbidity. In sub-Saharan countries, the problem of unsafe abortion arises more frequently [1, 2].

All mothers should not die due to unsafe abortion care, and therefore all women must have access to quality or safe abortion care. Nevertheless, approximately 13–18% of maternal deaths worldwide are due to complications caused by unsafe abortion care in developing countries [3]. The WHO estimates that each year about 22 million unsafe abortions are carried out, with over 98% taking place in poorer parts of the world. Additionally, approximately 5 million women are expected to experience complications following unsafe abortions. High-quality abortion care is key to reducing maternal morbidity and mortality related to unsafe abortion [3, 4].

The continuous improvement of service strategies should be a key goal of quality abortion care, which is an indispensable element of maintaining service quality and satisfying healthcare needs, women’s rights and freedoms [5]. Research has shown that clients who have received abortion care services but were not satisfied are often the ones who undermine the acceptability of legal abortion services among healthcare facilities, as a result of which women resort to unqualified providers and eventually to self-abortion. Therefore, this may induce abortion-related morbidity and maybe even mortality among women [6].

In order to improve the quality of facility-based abortion care, the healthcare provider should determine and provide for the woman’s needs. Client satisfaction is a key measure for evaluating the quality of healthcare service delivery that is also linked with other clinical outcome measures and medical malpractice claims [7]. Even though governments and healthcare workers have to do what they need to in every country, poor abortion care and unhappy clients are still problems all over the world today [8].

Clients who are satisfied with the treatment are more likely to be in compliance with the treatment, to assume their healthcare responsibilities, to arrive at appointments on time, to continue with the treatment, and to recommend it to family and friends. Where patient satisfaction results in four or five people hearing about the health facility’s services, patient dissatisfaction leads to 20 or more people complaining. Additionally, patient dissatisfaction is likely to lead to unsafe abortions [8–11]. As for the Ethiopian government, different programs, including comprehensive abortion care and post-abortion care, as well as compassionate, respectful care, were created in order to make the clients satisfied and improve abortion care. Such strategies are important as they can improve the quality of abortion care, provide interventions for a program to treat the complications resulting from improper abortion care, prevent unwanted pregnancies, and decrease the rate of repeat abortions. However, there are also challenges that need to be addressed by the government of Ethiopia and other stakeholders, such as reducing the risk of unsafe abortion, ensuring quality abortion care, and satisfying clients [12, 13].

Previous research highlights the influence of multiple factors, including patient-provider interaction quality as well as the information provided, individual-level, and social background features, on clients’ level of satisfaction with abortion care services. There are some other factors that have been used to highlight the issues of the abortion care services their clients received as well. These are type of procedure, women’s education level, facility type, intensity of pain experienced in management, patient’s choice of treatment method, access and availability of treatment option, health facility physical state and service provider’s attitude and perception [14–21]. According to literature from previous studies, the prevalence of client satisfaction in abortion care services was 90% in India [18], 91.6% in Pakistan [19], 92.6% in Guinea [20], and 26.9% in Dilla, Gedeo Ethiopia [21].

Though several studies have been conducted on patients’ satisfaction with abortion care services and associated factors in some parts of Ethiopia [21–30], none of them is generalizable or conclusive. Moreover, we still do not know the overall status of client satisfaction in abortion care or presumed factors in this regard. Therefore, this systematic review was conducted to determine the pooled prevalence of clients’ satisfaction and its associated factors among women who received abortion care in Ethiopia. At the end of the review, there were general recommendations and conclusions that would ensure the formulation of new policies and strategies towards quality abortion care and the improvement of abortion care services. It gives details of the prevalence of client satisfaction with abortion for all concerned bodies in Ethiopia. It goes a long way toward reducing maternal and neonatal death rates.

## Methods and materials

Systematic reviews and meta-analyses were conducted to estimate the overall prevalence of client satisfaction with abortion care services and its associated factors among women who received abortion care in Ethiopia.

### Search strategy

Databases such as Medline/PubMed, Web of Science, EMBASE, CINAHL, Med Nar, Google Scholar, Scopus, the Ethiopian University Repository Online, and the Cochrane Library were used to search for the studies included in this review. In addition, missing data were processed by contacting the corresponding authors. We checked the database https://www.crd.york.ac.uk/prospero and the Cochrane Library to ensure the review had not been done before and to avoid duplication. PROSPERO also registered this review with the registration number CRD42023460033. After confirming that there was no previously conducted review in Ethiopia, a comprehensive search strategy was developed using multiple Boolean operators through standard population comparison and exposure outcome (PEO) questions. The words “or” and “and” were used as search terms. The terms “abortion care,” “miscarriage care,” and “satisfaction AND factors affecting or influencing” OR client OR women OR mother AND Ethiopia, were searched using Boolean operators. Before being exported to the End Notes Library, all articles that are retrieved from the database are reviewed for titles and abstracts. These papers met the review criteria for inclusion in terms of titles and abstracts and were read in their entirety. A systematic review was used to evaluate the results of articles that were published in Ethiopia. Two authors (TG, EI) carried out the search strategy.

### Eligibility criteria

#### Inclusion and exclusion criteria

Studies that met the criteria for inclusion in this review assessed the prevalence of CS with abortion care services and its associated factors among women who received abortion care in Ethiopia. The studies included in this review were cross-sectional studies written in English. In addition, it included women who lived in Ethiopia. It also included studies conducted from 2013 to 2023. Studies conducted outside of Ethiopia, as well as studies with other study designs, were not included in this review.

### Data extraction and quality assessment

PRISMA was used to select and direct the selection of the articles for this review. The parameters used to extract the data include the author’s name, publication year, study location, the sample size for each study, and the study design. Using a Microsoft Excel spreadsheet, we gathered the necessary data from the accepted articles. Independently, two authors (TG and CK) extracted information from the supplementary documents. The studies that satisfied the eligibility requirements were included and summarized in the table after extensive agreement and discussion on data extraction and critical analysis using the Joanna Brings Institute Review Meta-analysis and Statistical Evaluation Tool (JBI-MASTER). JBI-MASTER identifies the studies and abstracts of the articles to decide whether they should be included. The quality of the included studies was evaluated independently by three reviewers (TG, EI, and CK) using JBI-MASTER. Any unclear information or disagreements between the two reviewers were resolved through deep discussion. The quality of the articles was assessed before choosing the final review. A quality assessment indicator score of seven or higher was considered low-risk for studies. The quality of the included studies assessed was more than 7, and there was low risk for all studies (Table 1).

**Table 1:** Critical appraisal results of eligible studies in this study on CS with abortion care in Ethiopia, 2023 (*n* = 10)

Tesfaye G et al. [26]	Q1	Q2	Q3	Q4	Q5	Q6	Q7	Q1	Q9	Total
Obsie GW et al. [21]	Y	Y	N	Y	Y	Y	Y	Y	Y	8
Gashaye TK et al. [22]	Y	Y	Y	N	Y	Y	Y	Y	Y	8
Sena BK et al. [23]	Y	Y	N	Y	Y	Y	Y	Y	Y	8
Oda T et al. [24]	Y	Y	Y	Y	Y	Y	Y	Y	Y	9
Guteta F et al. [25]	Y	N	Y	Y	Y	Y	Y	Y	Y	8
Balem D et al. [30]	Y	Y	Y	Y	Y	Y	Y	Y	Y	9
Ephrem T et al. [29]	Y	Y	Y	Y	Y	N	Y	Y	Y	8
Mossie C et al. [27]	Y	Y	Y	Y	Y	N	Y	Y	Y	8
Tetak E et al. [28]	Y	Y	Y	Y	Y	Y	Y	Y	Y	9

Y = Yes, N = No, U = Unclear; JBI critical appraisal checklist for studies reporting prevalence data: Q1 = was the sample frame appropriate to address the target population? Q2-Were study participants sampled appropriately? Q3-Was the sample size adequate? Q4-Were the study subjects and the setting described in detail? Q5-Was the data analysis conducted with sufficient coverage of the identified sample. Q6-Were the valid methods used for the identification of the condition? Q7-Was the condition measured in a standard, reliable way for all participants? Q8-Was there appropriate statistical analysis? Q9-Was the response rate adequate, and if not, was the low response rate managed appropriately

### Data processing and analysis

A Microsoft Excel spreadsheet was used to extract the data, and STATA version 14 was used to analyze the data. Using an analysis of random effects model synthesis, the overall prevalence of client satisfaction with abortion care services among women who received abortion care in Ethiopia was calculated. A meta-analysis was conducted to assess the influencing factors of client satisfaction with abortion care services. With the aid of a funnel chart and visual analysis, published trends were investigated. Testing for study heterogeneity was done using Cochrane Q-Static and I2. The quality of ANC services among women in the region was compared with an estimated prevalence using a subgroup analysis. A forest pilot graph was used to present a pooled prevalence of client satisfaction with abortion care services among women who received abortion care in Ethiopia.

## Result

### Identification and characteristics of the included studies

**Fig. 1:**
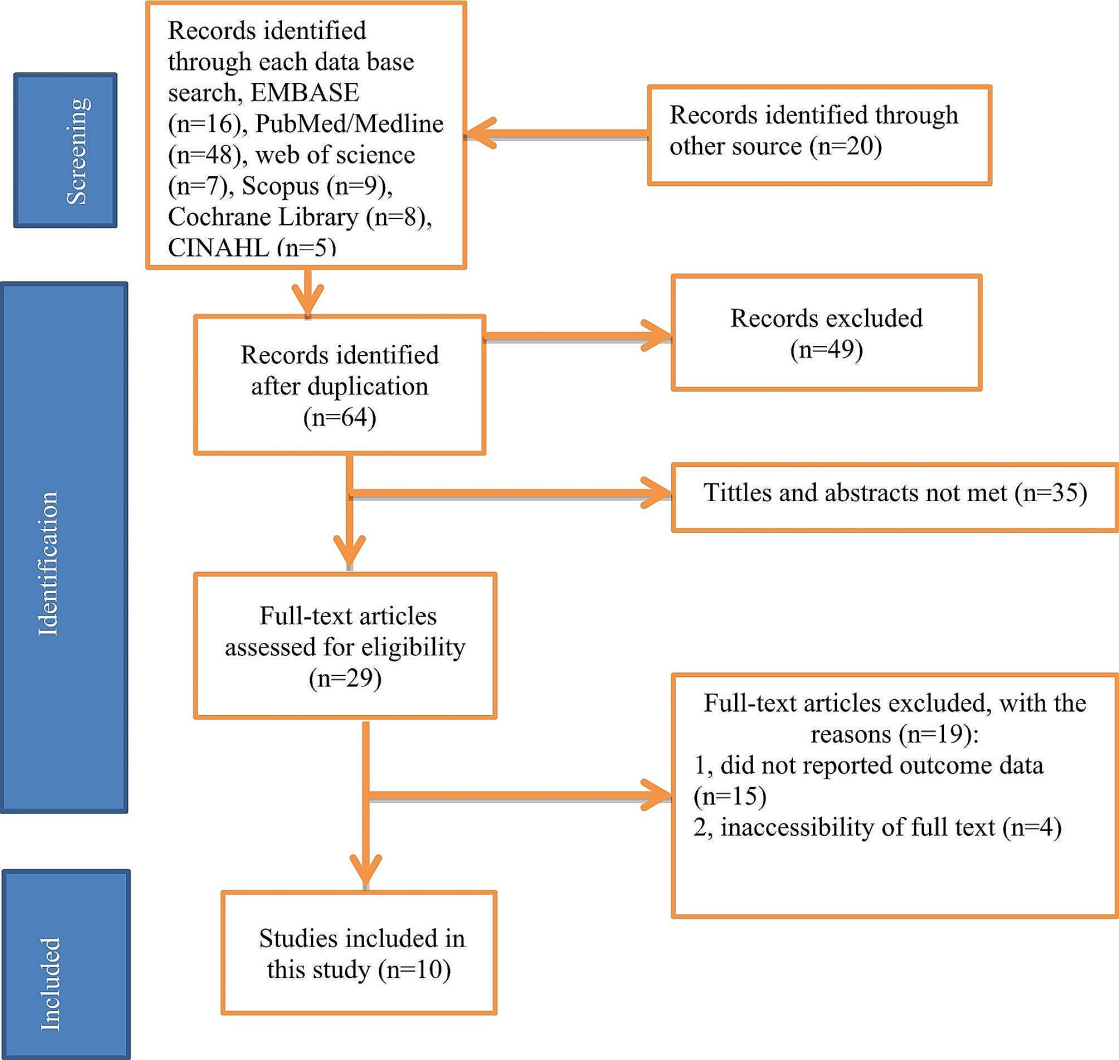
PRISMA Flow diagram of study selection for systematic review of CS with abortion care in Ethiopia, 2023(*n* = 10)

From January 1 to January 30, 2023, 113 articles were identified in the main electronic databases, and other suitable sources were searched. Of these identified articles, 49 were removed for duplication and 64 were reserved for further review. 35 studies were excluded because their abstracts and titles did not meet the requirements. Of the remaining 29 articles, 19 studies were excluded because they conflicted with the inclusion criteria set for this review. Finally, 10 studies that met the eligibility criteria were included in this review (Fig. 1).

A total of 10 articles with 2740 participants were included in this systematic review and meta-analysis. All included studies were cross-sectional studies, and sample sizes ranged from 78 [21] to 618 [22]. Regarding the regional distribution of included studies, three were from the Oromia region [23–25], two from the Southern Nations, Nationalities, and Peoples Region (SNNPR) [20, 26], two from Addis Ababa [27, 28], and one from the Amhara, Gambella, and Tigray regions [22, 29, 30] (Table 2).

**Table 2:** Study characteristics included in the systematic review of CS with abortion care in Ethiopia, 2023 (*n* = 10)

Author	Year	Region	Study area	Study design	Sample	Prevalence (%)
Tesfaye G et al. [26]	2013	SNNPR	Guragehe	Cross-section	422	84
Obsie GW et al. [21]	2020	SNNPR	Dilla	Cross-sectional	78	26.90
Gashaye TK et al. [22]	2022	Amhara	Northwest	Cross-sectional	618	25
Sena BK et al. [23]	2016	Oromia	Jimma	Cross-sectional	228	76.3
Oda T et al. [24]	2023	Oromia	Mojo	Cross-sectional	255	56.5
Guteta F et al. [25]	2022	Oromia	Nekemte	Cross-sectional	384	57.5
Balem D et al. [30]	2014	Tigray	Tigray	Cross-sectional	420	40.6
Ephrem T et al. [29]	2021	Gambella	Gambella	Cross-sectional	194	87.1
Mossie C et al. [27]	2016	Addis Ababa	Addis Ababa	Cross-sectional	450	60.48
Tatek T et al. [28]	2023	Addis Ababa	Addis Ababa	Cross-sectional	113	46

### Prevalence of CS with abortion care in the Ethiopia

**Fig. 2:**
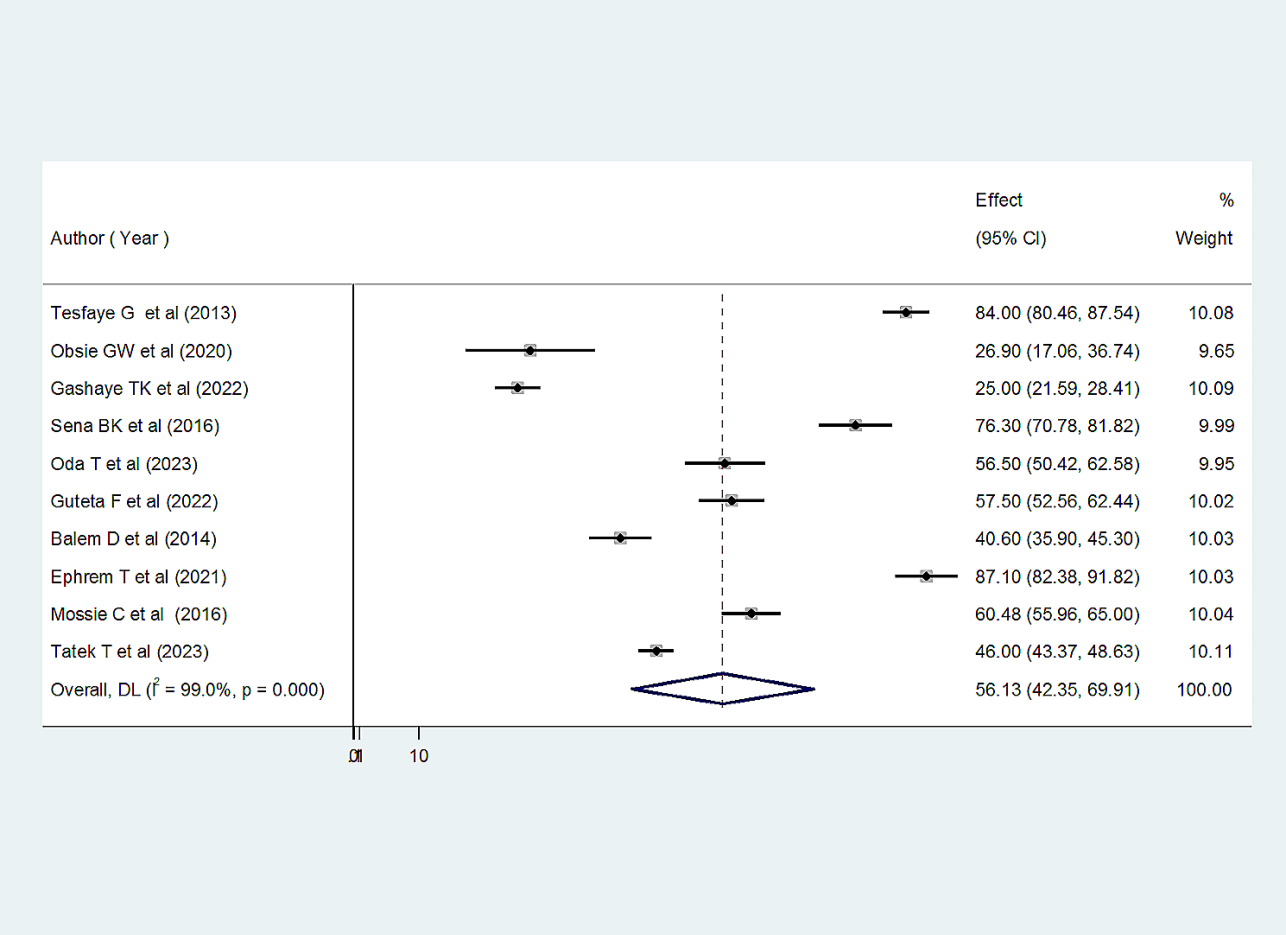
Forest plot showing the pooled prevalence of CS with abortion care in Ethiopia (*n* = 10)

Among the included studies, the status of CS with abortion care services among women in Ethiopia ranged from 25.00% [95% CI: 21.59, 28.40] [22] to 87.40% [95% CI: 82.38, 91.82] [29]. The overall status client satisfaction with abortion care in Ethiopia was 56.13% [95% CI (42.35; 69.91); I2 = 99.1%, *P* < 0.001] (Fig. 2).

### Subgroup analysis of client satisfaction with abortion care in Ethiopia

**Fig. 3:**
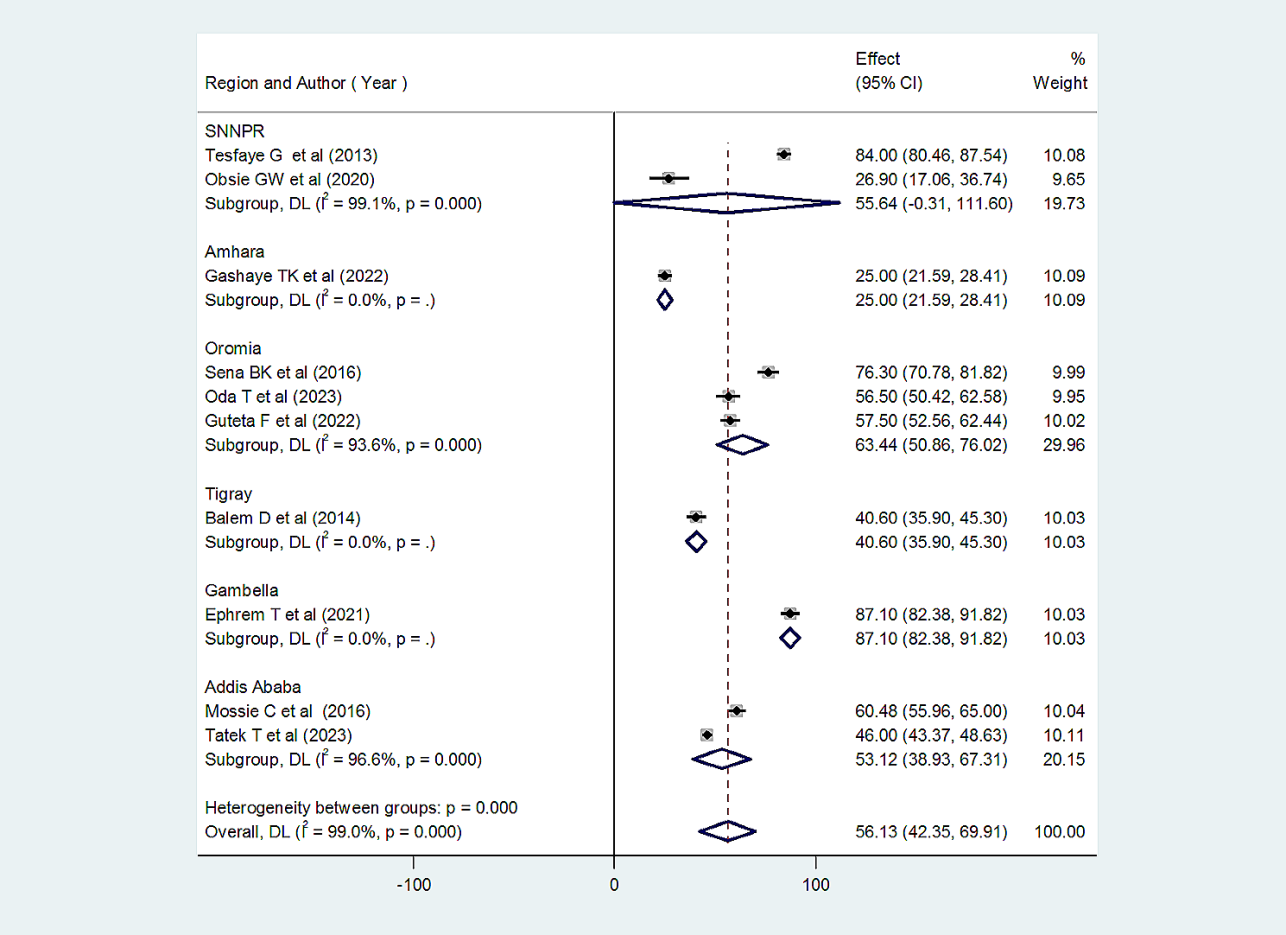
Subgroup analysis of CS with abortion care by region in Ethiopia (*n* = 10)

To investigate how the prevalence of client satisfaction with abortion care varies across regions, we have conducted a subgroup analysis. This analysis revealed that the highest client satisfaction with abortion care was found in the Gambella region with values of 87.40% [95% CI: 82.38, 91.82], followed by the Oromia region value of 63.44 (95% CI: 50.82, 76.02). The lowest values were observed in Amhara with 25.00% [95% CI: 21.59, 28.40] (Fig. 3).

### Outcome

The first interesting result of this systematic review and meta-analysis was the estimate of the pooled prevalence of client satisfaction with abortion care in Ethiopia. The review found that 56% of Ethiopian women were satisfied with the provision of abortion care. A second interesting finding is a factor related to client satisfaction. Results indicated that women’s educational level and type of procedure performed for the patient were significantly associated with client satisfaction.

### Heterogeneity and publication bias

**Fig. 4:**
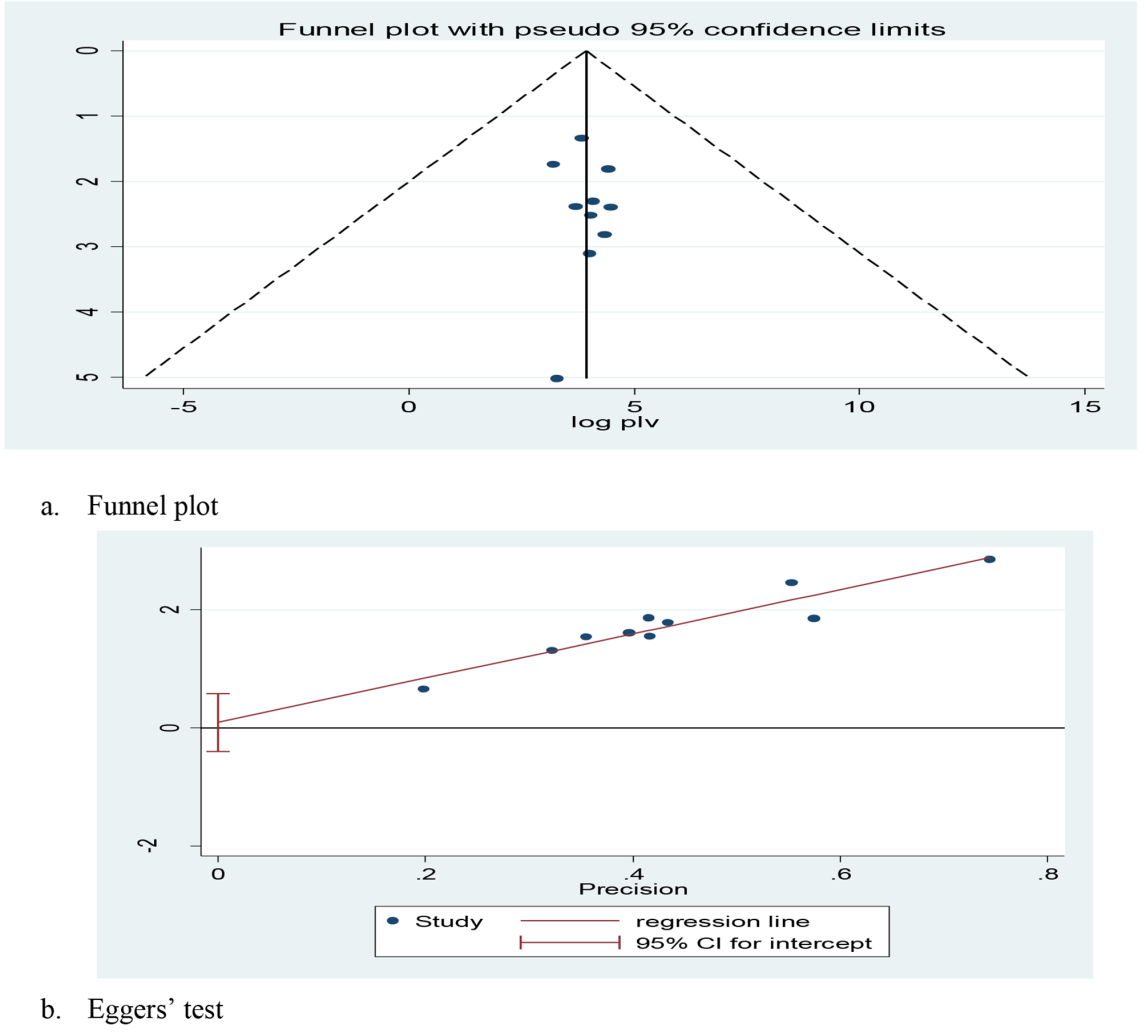
(**a**) funnel plot and (**b**) Eggers test (*P* = 0.679) of the review

To balance and compensate for the study heterogeneity, we performed subgroup analysis by regions. The results of the I2 test indicate considerable heterogeneity between studies (I2 = 99.00%, *p*-value < 0.001) (Fig. 2) and are controlled by a random effects model. The publication bias of the study was checked using Egger’s test and visual inspection of the funnel plots. The results of the funnel chart showed that the selected studies had a symmetrical distribution after inspection, indicating no bias among the included studies (Fig. 4a) and Eggers’ test (*P* = 0.679) (Fig. 4b). Lastly, we completed a sensitivity analysis by consecutively eliminating studies in order to understand the effect of a single study on the overall effect estimate. This finding confirmed that the elimination of one study not affected the prevalence of pool estimate (Table 3).

**Table 3:** Sensitivity analysis of systematic review and meta-analysis on the client satisfaction on abortion care and associated factors in Ethiopia

Study omitted	Estimate	[95% Conf. Interval]
Tesfaye G et al. (2013)	3.8491943	2.4067039 5.2916846
Obsie GW et al. (2020)	3.9431067	2.5947042 5.2915092
Gashaye TK et al. (2022)	4.0598927	2.6082108 5.5115743
Sena BK et al. (2016)	3.9059997	2.529145 5.2828541
Oda T et al. (2023)	3.9258871	2.5565569 5.2952175
Guteta F et al. (2022)	3.9216068	2.5340803 5.3091331
Balem D et al. (2014)	3.9511235	2.5576413 5.3446059
Ephrem T et al. (2021)	3.8843694	2.4914131 5.2773256
Mossie C et al. (2016)	3.9147	2.5162194 5.3131804
Tatek T et al. (2023)	3.9666963	2.4160569 5.5173354
Combined	3.93111	2.5951899 5.2670302

### Factors associated with the prevalence of client satisfaction with abortion care

In this review, two variables (type of procedure performed on women, women completed primary school) were significantly associated with the prevalence of client satisfaction in abortion care, while long waiting times, Women who had ever had an abortion, women who wanted a pregnancy, and women who had received adequate information before the procedure were not significantly associated with client satisfaction. This review showed a significant association between women’s education level and client satisfaction. Satisfaction levels for abortion care among participants who had attended primary school were 71% less likely as compared to other study participants [OR = 0.29, 95% CI [0.09, 0.48], *p* = 0.006, I2 = 76.9%)]. Women who got a surgical procedure had about 75% lower satisfaction rate compared to those who got a medical procedure (OR = 0.25, 95% CI [0.07, 0, 42], *p* = 0.041, I2 = 76.9%) (Table 4).

**Table 4:** Factors associated with prevalence of CS with abortion of the systematic review and meta-analysis in Ethiopia (*n* = 10)

Factors	OR	CI	I^2^	*P*-value	Significance level
Procedure type (Surgical)	0.25	[0.07, 0.42]	76.9%	0.041	Significance
Educational level (Primary)	0.29	[0.09, 0.48]	80.4%	0.006	Significance
Long waiting time (Yes)	1.19	[1.05, 1.34]	3.4%	0.309	Non-significance
Previous abortion history (Yes)	2.44	[0.17, 4.72]	00.0%	0.905	Non-significance
Wanted pregnancy (Yes)	1.56	[1.15, 2.13]	0.00%	0.585	Non-significance
Received adequate information before procedure (Yes)	0.06	[-0.11, 0.23]	65.6%	0.093	Non-significance

## Discussion

This systematic review and meta-analysis found that 56% of women who received abortion care in Ethiopia were satisfied with their abortion care. This result was lower than studies in India (90%) [18], Pakistan (91.6%) [19], and Guinea (92.6%) [20]. Such a discrepancy could possibly result from differences in geographical location, study period, policies, and/or strategies. In addition, the perceptions and attitudes of staff, the presence or absence of administrative support, organizational structure, study population, study setting, and methods may contribute to disparities. However, this review is higher than studies conducted in Tigray (25%), Amhara (40.6%), and southern Ethiopia (26.9%) [21, 22, 30]. Differences in the duration of the studies, study population, study setting, health care provider’s attitude, and methodologies could explain the discrepancies. This review also reports on CS in abortion care across the region. A subgroup analysis revealed that 84% of clients were satisfied with abortion care in Gambella, while the lowest percentage (25%) was in Amhara. It is possible that the reasons include issues concerning quality abortion care, study time, geographical location, health care professional attitudes and perceptions, and methodological issues.

This systematic and meta-analysis identified two factors associated with client satisfaction in abortion care, including the type of procedure performed on the patient and the educational level of the participants. Study participants who received surgical abortion care were 75% less satisfied with their abortion care than women who used medical abortion care. This study supports previous studies [23, 24]. The reason may be due to the painful and irritating nature of the surgical procedure. This can lead to lower patient satisfaction. Additionally, because surgery is intrusive, people may not receive adequate treatment from their health care provider or may arrive at a medical facility with very low expectations, fearing that it may affect their pregnancy [24].

Another factor associated with client satisfaction in abortion care was the woman’s educational level. Study participants who had completed elementary school were 71% less likely to be satisfied with their abortion care. This finding is consistent with those of previous studies [23, 24]. It could be if people learn more, they also expect more from their service providers to satisfy them. There is no communication gap between well-educated healthcare providers and patients. This means that uneducated customers may not ask about their interests in order to seek better service. Furthermore, less educated patients have less access to various medical facilities and social media, making them less well-informed [31, 32]. This can affect client’s expectations and satisfaction.

## Conclusion

This review found that 56% of Ethiopian women were satisfied with their abortion care. Therefore, enhancing the quality of abortion care in health facilities, understanding women’s expectations and perceptions, training health care providers, and strict monitoring, evaluation, and supervision of the quality of abortion care services are highly recommended to increase women’s satisfaction with abortion care by stakeholders like the Ethiopian government, non-governmental organizations, and all health facility top managers. The identified factors, such as the type of procedure for patients and women’s educational level, need to be improved through enhancing awareness about the type of procedure before conducting the surgical procedures and providing women with adequate health education about abortion care.

## Data Availability

the datasets generated and/or analyzed during the current study are available from the corresponding author upon reasonable request.
